# Transcriptional response of *Saccharomyces cerevisiae* to lactic acid enantiomers

**DOI:** 10.1007/s00253-023-12863-z

**Published:** 2024-01-13

**Authors:** Polina Drozdova, Anton Gurkov, Alexandra Saranchina, Anastasia Vlasevskaya, Elena Zolotovskaya, Elizaveta Indosova, Maxim Timofeyev, Ekaterina Borvinskaya

**Affiliations:** 1 Irkutsk State University, Karl-Marx Str. 1, Irkutsk, 664025 Russian Federation; 2 Baikal Research Centre, Rabochaya Str. 5V, Irkutsk, 664011 Russian Federation

**Keywords:** D-lactate, L-lactate, Lactic acid, Budding yeast, RNA-seq, Low pH stress

## Abstract

**Abstract:**

The model yeast, *Saccharomyces cerevisiae*, is a popular object for both fundamental and applied research, including the development of biosensors and industrial production of pharmaceutical compounds. However, despite multiple studies exploring *S. cerevisiae* transcriptional response to various substances, this response is unknown for some substances produced in yeast, such as D-lactic acid (DLA). Here, we explore the transcriptional response of the BY4742 strain to a wide range of DLA concentrations (from 0.05 to 45 mM), and compare it to the response to 45 mM L-lactic acid (LLA). We recorded a response to 5 and 45 mM DLA (125 and 113 differentially expressed genes (DEGs), respectively; > 50% shared) and a less pronounced response to 45 mM LLA (63 DEGs; > 30% shared with at least one DLA treatment). Our data did not reveal natural yeast promoters quantitatively sensing DLA but provide the first description of the transcriptome-wide response to DLA and enrich our understanding of the LLA response. Some DLA-activated genes were indeed related to lactate metabolism, as well as iron uptake and cell wall structure. Additional analyses showed that at least some of these genes were activated only by acidic form of DLA but not its salt, revealing the role of pH. The list of LLA-responsive genes was similar to those published previously and also included iron uptake and cell wall genes, as well as genes responding to other weak acids. These data might be instrumental for optimization of lactate production in yeast and yeast co-cultivation with lactic acid bacteria.

**Key points:**

*• We present the first dataset on yeast transcriptional response to DLA.*

*• Differential gene expression was correlated with yeast growth inhibition.*

*• The transcriptome response to DLA was richer in comparison to LLA.*

**Supplementary Information:**

The online version contains supplementary material available at 10.1007/s00253-023-12863-z.

## Introduction

The model yeast species *Saccharomyces cerevisiae* is used in many fundamental and applied research applications, such as production of many industrially relevant compounds (Krivoruchko and Nielsen 2015) and as biosensors to various substances, for example, heavy metals or estrogens (Martin-Yken 2020). The compounds produced in yeast include D-lactic acid (DLA) (Baek et al. 2016), which is used for production of stereocomplex type poly-lactic acid, a promising biodegradable polymer (de Albuquerque et al. 2021). Genetically engineered yeast strains produce up to 112 g/L (1.24 M) of DLA in neutralizing conditions or over 53.2 g/L (0.59 M) of this substance without neutralizing agents (Ishida et al. 2006, 2011; Baek et al. 2016; Yamada et al. 2017; Mitsui et al. 2020). Efficient sensing systems for DLA would also be relevant in medicine, as D-lactic acidosis is a rare but serious neurologic condition specific to individuals with short bowel syndrome (Kowlgi and Chhabra 2015; Petersen 2005).

However, a systematic evaluation of yeast cell response is scarce not only for DLA but even for its incomparably more common enantiomer L-lactic acid (LLA). The response to LLA has been studied in several yeast strains on the levels of viability or growth rate reduction, as well as transcription. Specifically, it was found that lactic acid (LA) in industrially relevant concentrations of 90 or even 280 mM LA (presumably LLA) had a rather limited effect on the metabolism of *S. cerevisiae* in thermostat cultures but did affect the energy status of the cell by provoking a reduction in the ATP content (Thomsson and Larsson 2006). Another study estimated the minimal inhibitory concentration of LA (presumably LLA) as 2.5% w/v (278 mM), while 0.2% began to stress the cells (Narendranath et al. 2001). There are also two studies on the transcriptional effect of LLA. One of them dealt with LLA in comparison with acetic and hydrochloric acids with DNA microarrays in shake flask cultures. The authors found that these organic acids triggered relatively similar gene expression perturbations and affected cell wall and metal metabolism; the latter was intermediated by the Atp1p transcription factor (Kawahata et al. 2006). Another study, using chemostat cultures, also found iron metabolism remodeling; it was very pronounced at pH 5 and 500 mM LLA and much less severe at pH 3 and 900 mM LLA (Abbott et al. 2008). To the best of our knowledge, there is no published data on the effect of DLA on yeast transcriptome or proteome.

In this study, we evaluated the transcriptional response of *S. cerevisiae* to varying concentrations of DLA (from 0.05 mM to 45 mM) and 45 mM of LLA in order to check if this model species possessed any promoters that would quantitatively respond to DLA but not LLA and could thus be promising for designing a yeast-based stereo-specific biosensor to lactic acid. Such transcriptome-based approach has already been successfully applied to find a 1-butanol sensing promoter in *S. cerevisiae* (Shi et al. 2017). In addition, we aimed at enriching the data on transcriptional response to DLA in comparison to LLA. We found that the concentrations of DLA of 0.05 or 0.5 mM did not trigger any changes in gene expression compared to the control samples. The genes activated in response to 5 mM DLA were enriched in those controlling cell wall organization, while the genes upregulated upon 45 mM DLA treatment included several genes functioning in lactate metabolism and iron uptake. Finally, the genes responding to LLA contained many genes known to respond to this and other weak acids.

## Materials and methods

### Yeast cultivation

The yeast strain used for this work was BY4742 (*MATα his3Δ1 leu2Δ0 lys2Δ0 ura3Δ0*; Baker Brachmann et al. 1998). For the experimental exposures, overnight suspension cultures were inoculated from an independent BY4742 colony on solid YPD medium (1% yeast extract, 2% peptone, 2% D-glucose, and 2% agar) and grown in synthetic medium containing 0.67% (w/v) yeast nitrogen base, 2% (w/v) glucose, 150 mM NaCl, 20 mg/L L-histidine HCl, 100 mg/L L-leucine, 30 mg/L L-lysine HCl, and 20 mg/L uracil at 28 °C with orbital shaking (120 rpm). The addition of NaCl had the purpose of mimicking the conditions in the blood plasma for further use in sensor applications. The next day, approximately 1.5 optical density units of each culture were collected by centrifugation (1700 × g for 5 min at room temperature) and resuspended in 3 ml of the same media (control) or media with lactic acid. OD600 was recorded at the beginning of the exposure and in 3–4 h. After this time, the experimental cultures were collected by centrifugation and frozen at -80 °C for RNA extraction. The relative growth rate was calculated as the difference between logarithmic (base 2) final OD600 and initial OD600 divided over incubation time in hours. This experiment was performed in total seven times with independent suspension cultures with 0.05, 0.5, 5, or 45 mM DLA, or 45 mM LLA and a control medium; three of these replicates were used for the RNA sequencing. Moreover, similar exposures were performed with eight independent suspension cultures with 45 mM DLA, 45 mM sodium D-lactate (DLS), and a control medium; five of these replicates were used for qPCR testing.

### RNA extraction, sequencing, and quantitative PCR (qPCR)

RNA extraction, library construction, and sequencing were performed by the CeGaT company (Tübingen, Germany). RNA isolation was performed with the RNeasy kit (Qiagen, Hilden, Germany) according to manual (RNeasy Mini Handbook) with slight modifications. Cells were homogenized by mechanical disruption. After the addition of RLT buffer (Qiagen, Hilden, Germany) and glass beads, the samples were vortexed three times for 3 min. After each vortexing step, the samples were cooled on ice. Then, the lysate was centrifuged for 2 min at maximum speed, and the supernatant was transferred to new microcentrifuge tubes, combined with the same volume of 70% ethanol and mixed by pipetting. Sequencing libraries were prepared with the TruSeq Stranded mRNA kit (Illumina Inc., CA, USA) and sequenced at 2 × 100 bp with a NovaSeq 6000 (Illumina Inc., USA). Demultiplexing of the sequencing reads was performed with Illumina bcl2fastq v2.20, and adapters were trimmed with Skewer v 0.2.2 (Jiang et al. 2014). For each sample, between 2.8 and 7.4 Gb were sequenced.

RNA extraction for qPCR-based gene expression analysis was performed with the RNASwift method according to the original protocol (Nwokeoji et al. 2016) using GeneJET spin columns (Thermo Scientific, Waltham, MA, USA) and buffers provided with the RNeasy kit (Qiagen, Hilden, Germany) at the last step. RNA purification was performed according to the recommendation of the buffer manufacturer. Then, RNA was treated with RapidOut DNA removal kit (Thermo Scientific, Waltham, MA, USA) to remove residual genomic DNA. Then, RNA concentration was measured with the Nano-300 (Allsheng, Hangzhou, China) micro-spectrophotometer, and approximately 60–70 ng of DNA-free RNA was used for reverse transcription, which was performed with RevertAid reverse transcriptase and the corresponding buffer (Thermo Scientific, Waltham, MA, USA), RiboLock RNase inhibitor (Thermo Scientific, Waltham, MA, USA), dNTPs and Oligo(dT)18 primers (Thermo Scientific, Waltham, MA, USA) according to the recommendation of the enzyme manufacturer. Then, 1 μL of the resulting cDNA of the resulting solution was used for 10-μL qPCR. The amplification was performed using a StepOne Plus instrument (Thermo Scientific, Massachusetts, USA) with the 5X qPCRmix-HS SYBR Hi-Rox (Evrogen, Moscow, Russia) and primers (5 pmol each) specific for the following genes: *ACT1* and *CDC19* used as reference genes; *AQR1*; *DLD3*; *FIT2*; and *YPS3*. Primer sequences (Supplemental Table S1) for *ACT1* and *CDC19* were taken from the work by Cankorur-Cetinkaya et al. (2012); the other primer pairs were designed with NCBI Primer Blast (Ye et al. 2012). Amplification efficiency was tested for each primer pair with serial dilutions of a control cDNA sample and lied in the range of 88–100% (Supplemental Table S1).

### Data analysis and availability

Quality control of raw data was performed with FastQC v0.11.9 and summarized with MultiQC v1.13 (Ewels et al. 2016). The R64-1–1 release of *S. cerevisiae* strain S288C genome (Engel et al. 2014) was downloaded from Ensembl (Cunningham et al. 2022) release 108 and used as a reference. The reads were aligned to the genome with hisat2 (Kim et al. 2019) v2.2.1, sorted with samtools (Li et al. 2009) v1.9 and quantified with featureCounts (Liao et al. 2014) from subread v2.0.4.

The resulting count table was further processed with the DESeq2 (Love et al. 2014) v1.34.0 for R (R Core Team 2022) v4.1.2 to compare expression levels. The figures were prepared using the ggplot2 (Wickham 2016) v3.4.2, enhancedVolcano (Blighe et al. 2023) v1.12.0 and DEGReport (Pantano et al. 2023) v1.30.3 packages for R. The data from the Abbott et al. (2008) manuscript, which were used for comparison, were downloaded from the NCBI GEO database (accession number GSE10066) with the script generated by the GEO2R service (Edgar et al. 2002), which utilizes the GEOquery (Davis and Meltzer 2007) 2.62.2, limma (Ritchie et al. 2015) 3.50.3, and DESeq2 packages for R. Gene ontology (GO) term and publication enrichment analyses were performed with YeastMine (Balakrishnan et al. 2012; Cherry et al. 2012) using the database of 1 Apr 2023.

All the code used is available at GitHub (Drozdova 2023). The raw and processed RNA sequencing data are also available from the NCBI GEO repository under the accession number GSE231937.

## Results

### Overview of transcriptional response to lactic acid enantiomers

**Fig. 1:**
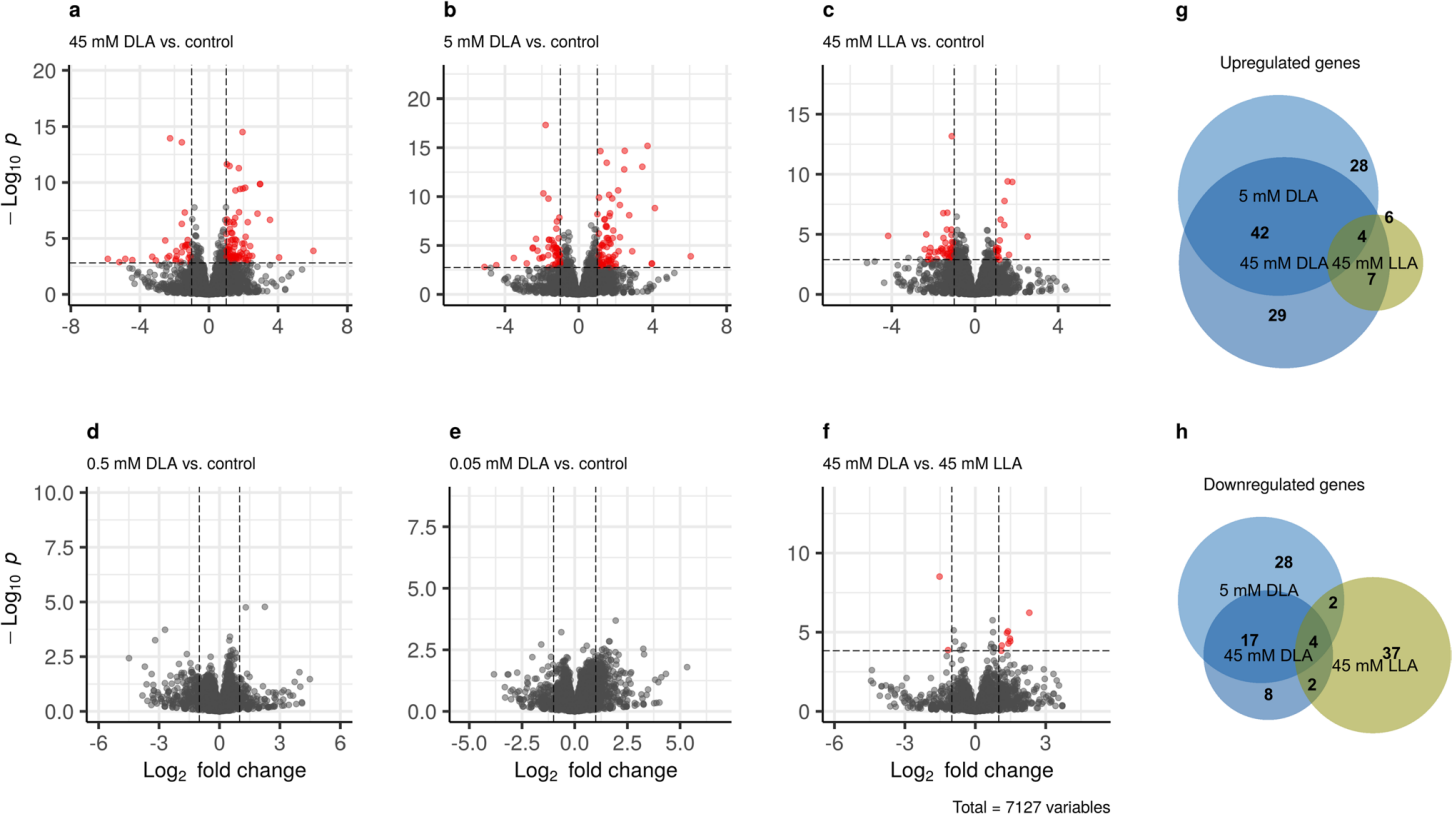
Overview of gene expression changes in all comparisons. **a**–**f** show volcano plots for major comparisons with differentially expressed genes (absolute log_2_ fold change > 1 and adjusted *p*-value < 0.05) are indicated by red dots (each dot corresponds to one gene). **g**, **h** show intersections of the lists of genes upregulated and downregulated, respectively, in response to different treatments, represented as Venn diagrams. Full expression data are available in Supplemental Table S3 and from the GEO database (GSE231937)

The performed analysis revealed differentially expressed genes (hereafter DEGs; absolute log2 fold change > 1 and adjusted *p*-value < 0.05) only in the case of the two highest DLA concentrations (5 mM and 45 mM), as well as in the case of 45 mM LLA (Fig. 1a–c). The concentrations of 0.5 mM DLA and below did not produce any significant transcriptional response (Fig. 1d, e). Overall, the presence/absence of DEGs correlated with the growth inhibition: whenever growth was inhibited, we recorded differential expression (Supplemental Fig. S1; Supplemental Table S2). Finally, there were 10 genes that were differentially expressed between the maximal concentration of DLA and the same concentration of LLA (see below in the section “Transcriptional response to LLA and search for DLA-specific genes”).

Furthermore, we functionally characterized DEG lists with gene ontology terms using YeastMine (Table 1). We found that the genes upregulated in response to 45 mM were enriched with those participating in lactate biosynthesis and metabolism (these genes will be characterized in detail below) and siderophore transport. The genes upregulated in response to a lower concentration of DLA (5 mM) were connected to the cell wall, while those downregulated in these conditions contained three genes regulating leucine biosynthesis. In the case of 45 mM LLA, we only found one very general enriched GO term for downregulated genes, generation of precursor metabolites, and energy.

**Table 1:** Significantly (*p* < 0.05) overrepresented GO terms

45 mM DLA vs. control	
Upregulated (82 genes)	Downregulated (31 genes)
Lactate metabolic process (GO:0006089)	No enrichment found
Siderophore transport (GO:0015891)	
Lactate biosynthetic process (GO:0019249)	
5 mM DLA vs. control	
Upregulated (74 genes)	Downregulated (51 genes)
Cell wall organization or biogenesis (GO:0071554)	Leucine biosynthetic process (GO:0009098)
External encapsulating structure organization (GO:0045229)	
Cell wall organization (GO:0071555)	
Fungal-type cell wall organization or biogenesis (GO:0071852)	
Fungal-type cell wall organization (GO:0031505)	
Cell wall biogenesis (GO:0042546)	
45 mM LLA vs. control	
Upregulated (17 genes)	Downregulated (45 genes)
No enrichment found	Generation of precursor metabolites and energy (GO:0006091)
45 mM DLA vs. 45 mM LLA	
Upregulated (8 genes)	Downregulated (2 genes)
No enrichment found	No enrichment found

### Transcriptional response to DLA

**Fig. 2:**
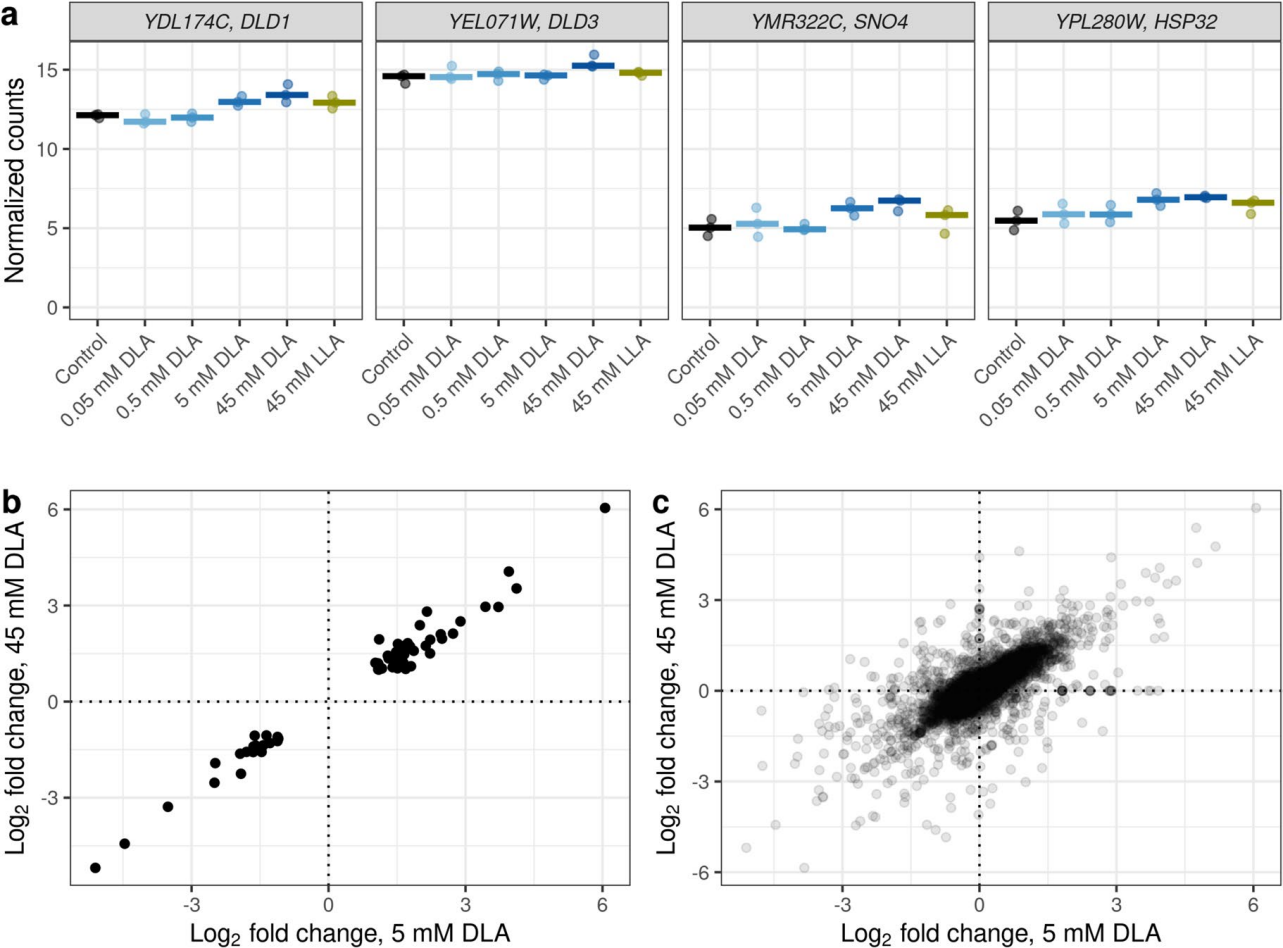
Overview of transcriptional response to DLA. Shown are **a** logarithmic (base 2) normalized expression counts of the genes DE in response to 45 mM DLA and annotated with the GO terms “lactate metabolic process” or “lactate biosynthesis process” (ORF (open reading frame) symbols and gene names shown in the titles of the panels), as well as the correlation between expression changes in response to 5/45 mM DLA of shared DEGs (**b**) and all genes (**c**). Pearson’s correlation coefficient = 0.99 for (**b**) and 0.72 for (**c**)

In the case of the highest concentration of DLA, we found significant enrichment of two GO terms connected to lactate (Table 1). The DEGs annotated with the terms “lactate metabolic process” (GO:0006089) and “lactate biosynthetic process” (GO:0019249) largely overlapped and contained *DLD1* (YDL174C), *DLD3* (YEL071W), *SNO4* (YMR322C), and *HSP32* (YPL280W). The former two genes indeed encode D-lactate dehydrogenases, mitochondrial Dld1 and cytoplasmic Dld3 (Pallotta 2012); according to the literature, Dld3 can also oxidize D-2-hydroxyglutarate to α-ketoglutarate (Becker-Kettern et al. 2016). The latter two are specific small chaperones (Gong et al. 2009). Unfortunately, none of these four genes was both DLA-specific and quantitatively responding to DLA (Fig. 2a).

In the case of 5 mM DLA, the main groups of upregulated genes were those associated with cell wall biogenesis (Table 1; Supplemental Table S4). This effect is not probably specific for DLA, as a similar effect was found for different organic acids (Kawahata et al. 2006). These genes also reacted to 45 mM DLA, even though less strongly (Supplemental Fig. S2). Overall, the transcriptional responses to 5 mM DLA and 45 mM DLA correlated quite well (Fig. 2b, c).

### Transcriptional response to LLA and search for DLA-specific genes

We found fewer DEGs in response to LLA in comparison to DLA; moreover, there were no overrepresented GO terms for upregulated genes and only a rather vague term “generation of precursor metabolites and energy” in the case of downregulated genes. However, the lists of genes differentially expressed in response to LLA were associated with many (over 20) publications enriched in some of these genes (Supplemental Table S4). Impressively, four of the six manuscripts enriched in LLA-upregulated genes dealt with the Haa1 transcription factor, which was indeed shown to mediate the adaptation of yeast cells to lactic and other weak acids (Fernandes et al. 2005; Mira et al. 2010, 2011; Sugiyama et al. 2014).

**Fig. 3:**
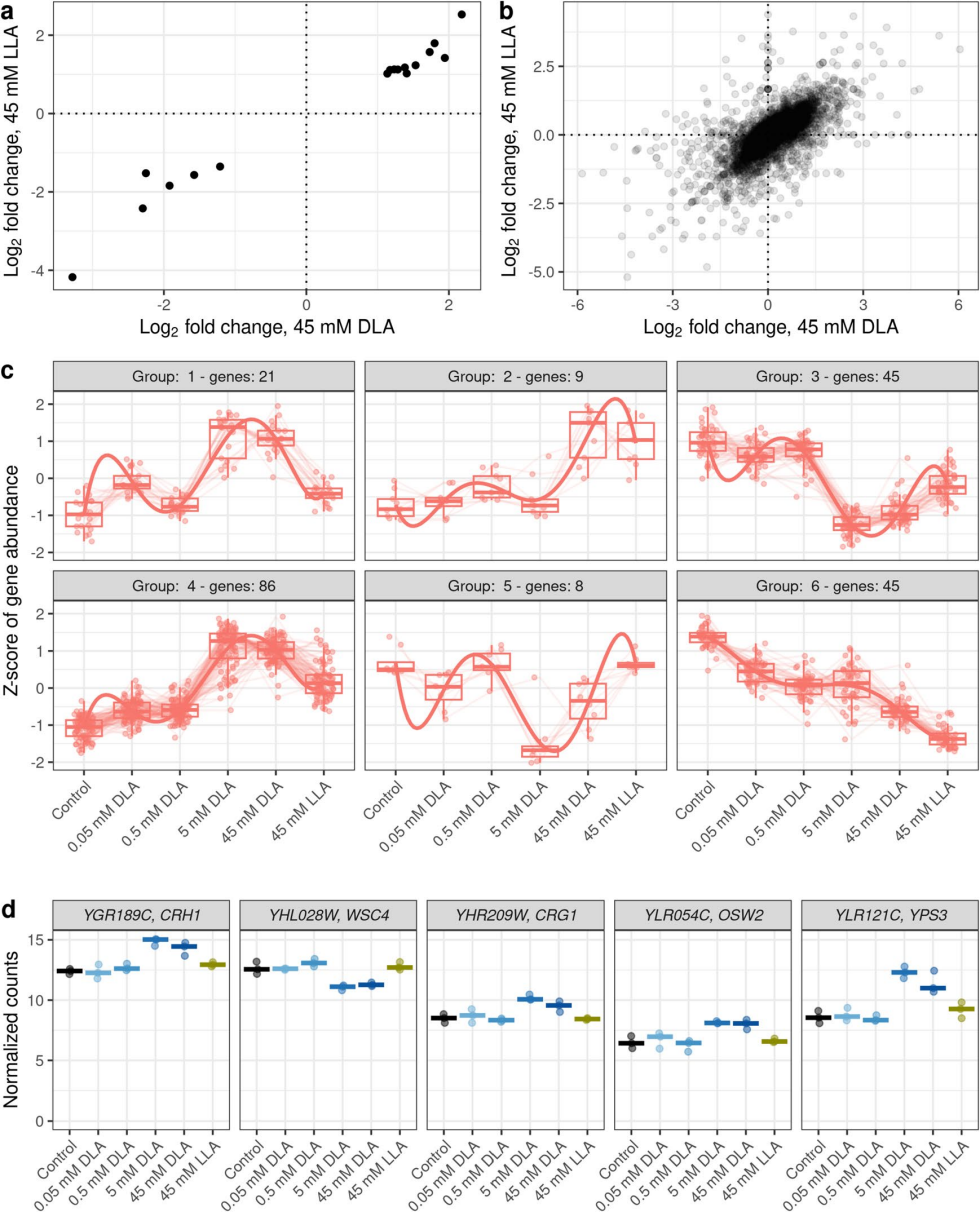
Comparison of transcriptional responses to LLA and DLA shows significant similarities and reveals several candidate qualitative DLA sensor genes. Correlation between expression changes in response to 45 mM DLA or LLA of shared DEGs (**a**) and all genes (**b**). Pearson’s correlation coefficient = 0.98 for **a** and 0.65 for **b**. **c** Clustering of expression profiles does not reveal any groups with quantitative response to DLA. **d** Expression profiles of potential qualitative sensors, genes responding to DLA but not LLA. The vertical axis shows logarithmic (base 2) normalized expression counts of the genes

Generally, the changes triggered by LLA correlated well to the changes observed in response to the same concentration of DLA (Fig. 3a, b). In order to reveal if there were any genes that quantitatively responded to DLA and did not respond to LLA, we performed a clustering analysis of 214 genes that were differentially expressed in at least one condition. Within the obtained six clusters, none had the desired pattern for a DLA sensor, i.e., monotonous increase or decrease in response to DLA and absence or very slight response to LLA (Fig. 3c). It is worth mentioning that the first cluster featured genes which were seemingly affected more by the high concentrations of DLA than by LLA, but in fact, the changes were very subtle (Supplemental Fig. S3).

In addition, we analyzed the genes differentially expressed between 45 mM DLA and 45 mM LLA (Fig. 1f). There were ten such genes, YHL028W (*WSC4*), YLR054C (*OSW2*), YLR121C (*YPS3*), YGR189C (*CRH1*), YGR146C (*ECL1*), YGL255W (*ZRT1*), YHR209W (*CRG1*), YLR205C (*HMX1*), YKR091W (*SRL3*), and YCR005C (*CIT2*). Some of these genes (*WSC4*, *YPS3*, *CHR1*, and *OSW2*) regulate cell wall assembly. Wsc4, Yps3, and Crh1 have functions in maintaining cell wall integrity (Verna et al. 1997; Krysan et al. 2005; Cabib et al. 2007). Osw2 is a protein of unknown function, which is putatively involved in spore wall assembly (Coluccio et al. 2004). Several genes (*ZRT1*, *ECL1*, and *HMX1*) are implicated in metal transport. Zrt1 is a zinc transporter (Zhao and Eide 1996). Ecl1 is a protein of unknown function upregulated by overexpression of the other iron deprivation-responding transcription factor, Aft2 (Rutherford et al. 2003). *HMX1* encodes a heme oxygenase, and expression of this gene is regulated by the iron deprivation-responding transcription factor Aft1 (Protchenko and Philpott 2003). Finally, the link between some of the genes and LA stress was unclear. Crg1 is a small molecule methyltransferase regulating lipid homeostasis in response to a drug cantharidin (Lissina et al. 2011), Cit2 is a citrate synthase (Kim et al. 1986), and Srl3 (or Whi7) participates in cell cycle regulation (Gomar-Alba et al. 2017). Of these genes, five reacted to both 5 mM and 45 mM DLA but not to 45 mM LLA (Fig. 3d). These could be candidates for qualitative sensors for DLA but required further exploration.

### Neutralization compensates for the effect of the DLA on growth rate and transcription of selected genes

**Fig. 4:**
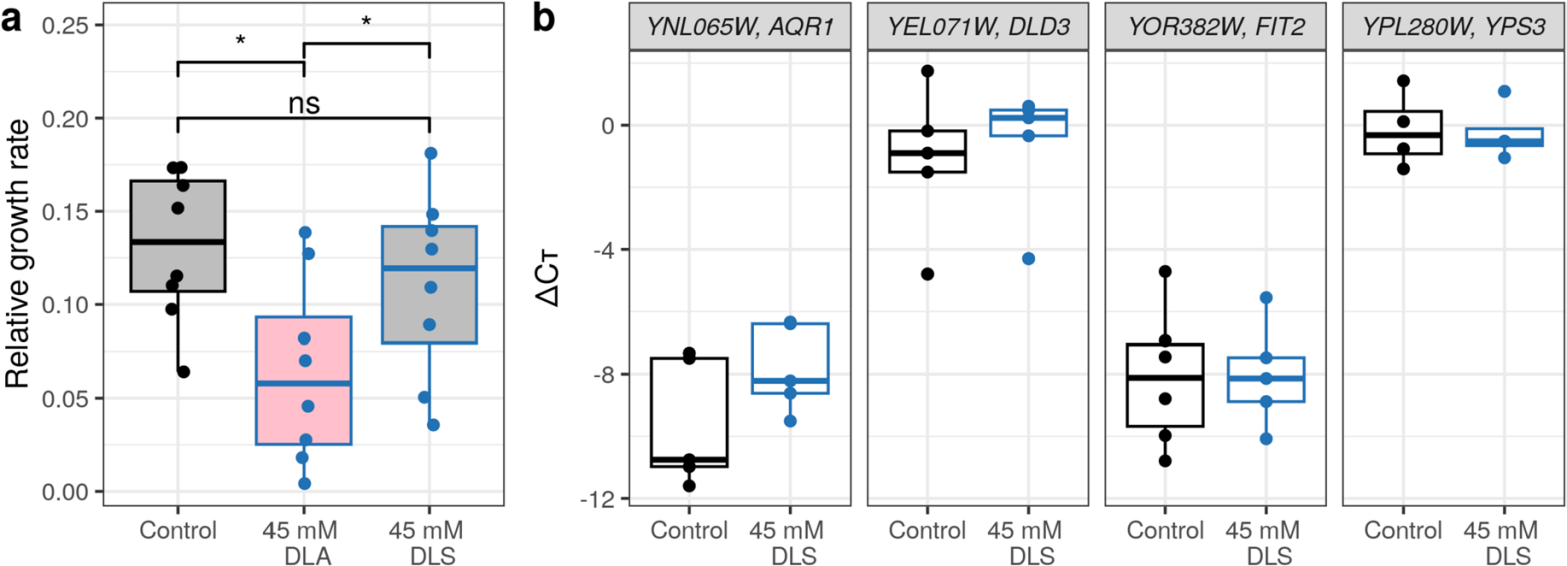
Addition of 45 mM sodium D-lactate compensates for the growth defect caused by 45 mM DLA treatment (**a**) and does not trigger changes in the expression of selective DLA-responsive genes (**b**). **a** Relative growth rate (the difference between logarithmic (base 2) final OD600 and initial OD600 divided over incubation time in hours) of cultures incubated for 3 h in the media with 45 mM DLA or DLS. *p < 0.05; ns, not significant (paired pairwise Wilcoxon rank sum test). **b** Expression levels of the *AQR1*, *DLD3*, *FIT2*, and *YPS3* genes relative to the geometric mean of the reference genes *ACT1* and *CDC19* measured with quantitative PCR. Raw qPCR data are presented in Supplemental Table S5

During all previous analyses, we found several groups of genes that reacted to one (45 mM) or two (5 and 45 mM) concentrations of DLA, and we also found that these treatments slowed down yeast growth (Supplemental Fig. S1). In order to check if the slow growth was caused by the lower pH values, we performed the same treatment but with 45 mM sodium D-lactate (DLS) obtained with addition of the same amount of NaOH (the concentration of additional NaCl in the media was adjusted to sum up to 150 mM) and indeed observed compensation of the growth defect (Fig. 4a). Thus, the observed slow growth in yeast treated with 45 mM DLA is explained by pH shift and not by the influence of the D-lactate ion.

Moreover, we checked if the expression of several genes that we found to be DLA-responsive changed in response to DLS. For this analysis, we chose the *AQR1*, *DLD3*, and *FIT2* genes, which were activated to 45 mM DLA but did not respond to 5 mM DLA, as well as the *YPS3* gene that responded to both concentrations of DLA. These genes belong to different functional groups. *AQR1* (YNL065W) encodes a membrane protein from the major facilitator superfamily, which provides the cells with resistance to short-chain monocarboxylic acids (Tenreiro et al. 2002). *FIT2* (YOR382W) is a cell wall mannoprotein involved in the siderophore transport (Protchenko et al. 2001), and we chose it as a representative of a larger group of iron uptake-related proteins (Table 1). *DLD3* (YEL071W) codes for a protein with D-lactate dehydrogenase activity in vitro, but there is evidence that in vivo it contributes to D-lactate synthesis (Chelstowska et al. 1999; Becker-Kettern et al. 2016). Finally, *YPS3* (YLR121C) encodes an aspartic protease required for cell wall integrity (Olsen et al. 1999), and we chose it as a representative of the cell wall integrity-related genes and also because it had strong changes in expression in response to both DLA concentrations but not to LLA (Fig. 3d) and thus could act as qualitative DLA sensor. However, we found that the relative expression levels of all of these genes were very similar in the control samples and in those treated with 45 mM DLS. Taken together, our data suggest that the expression changes in response to DLA were mostly triggered by the change in the pH value.

## Discussion

In this study, we explored the transcriptional response of *S. cerevisiae* to LA enantiomers. One of the goals of this work was to find genes that would specifically and quantitatively respond to DLA but not to LLA. We did not find such genes. In general, we found that the response to high concentration of DLA and LLA was quite similar and even more pronounced to DLA than to LLA. It is possible that the reason for this difference is that LLA is metabolized faster. There was no response to the concentrations of DLA of 0.5 mM and below. It is possible that higher DLA concentrations (> 50 mM) or longer exposures (about a day) could have made the effect more pronounced and reveal differentially expressed genes. However, higher concentrations would be outside the range of DLA concentrations typically found in biological fluids. While normal levels of LLA in mammal blood plasma are about 1–2 mM, the levels of DLA are about two orders of magnitude lower, 0.01–0.07 mM (Ewaschuk et al. 2005). In general, the levels of < 0.2 mM are considered normal (Zhang et al. 2003). The most well-known condition leading to D-lactic acidosis in human is short bowel syndrome, during which plasma DLA levels may reach millimolar concentrations (Zhang et al. 2003; Yilmaz et al. 2018). Similar symptoms in ruminants appear upon elevated carbohydrate level in their diet (Lorenz and Gentile 2014) and may lead to DLA plasma levels as high as about 25 mM (Ewaschuk et al. 2005). Thus, an ideal biosensor for DLA should be sensitive at 0.1–5 mM concentration range. Similarly, the need to use longer exposures of sensor yeast would also hinder its usage in biosensor applications. So, we find it unlikely that a quantitative yeast sensor to DLA may be constructed based on the native yeast transcriptional networks, but the possibility of integrating a heterologous cassette remains open. Such a cassette has been described for *Pseudomonas*, but its sensitivity starts from about 20 mM DLA (Singh et al. 2019), which is also far from ideal for monitoring DLA levels in biological fluids.

Importantly, we present the first dataset on yeast transcriptional response to DLA. Generally, we found that if the particular concentration did not inhibit growth, we did not see a transcriptional response either. This result is very similar to the study, in which the authors compared the transcriptome-wide responses to different alcohols in search for 1-butanol sensor and found that the samples treated with ethanol, which did not cause significant growth inhibition, clustered with the control samples, while samples treated with 1-butanol and 1-propanol, which were much more toxic, clustered separately (Shi et al. 2017). Intriguingly, the response to 5 mM and 45 mM was seemingly different if judging by enriched GO terms (5 mM DLA caused overexpression of cell wall-related genes, while 45 mM led to increased expression of lactate metabolism and siderophore transport genes), but the transcriptional profiles in response to 5 mM and 45 mM DLA were highly correlated (Fig. 2c), and more than half of the DEGs were shared between the two comparisons (Fig. 1g, h).

In general, the groups of cell-wall related genes and genes of iron/siderophore uptake have already been described to respond to weak acid stress (Kawahata et al. 2006; Abbott et al. 2007, 2008), so our findings fully corroborate the previously published data but at the same time suggest that this response might be pH-dependent rather than specific for the lactic acid. However, comparison of four weak acids, benzoate, sorbate, acetate, and propionate, showed that the transcriptional responses were largely specific (Abbott et al. 2007), proving that the response to pH was not the only reason for gene expression changes. We have checked this hypothesis for the *FIT2* gene, which codes for cell wall mannoprotein involved in the siderophore transport (Protchenko et al. 2001) as a representative of the iron/siderophore uptake functional group and *YPS3*, the gene encoding an aspartic protease required for cell wall integrity (Olsen et al. 1999), as a representative of the cell wall genes group. Both genes did not respond to D-lactate treatment if pH was compensated (Fig. 4b). Moreover, in this experiment we also measured the expression levels of the *AQR1* gene. It encodes a membrane protein from the major facilitator superfamily, which provides the cells with resistance to short-chain monocarboxylic acids (Tenreiro et al. 2002). We found that *AQR1* also only responded to DLA at low pH. Interestingly, the authors of the original manuscript noted that the expression of this gene was not stimulated by weak acid stress (Tenreiro et al. 2002). We found that it was upregulated in response to 45 mM DLA, while Abbott et al. (2007) found it as a common gene downregulated in response to the four weak organic acids they tested. Aqr1 has not been shown to have a role in lactic acid transport, but it acts as a lactic acid exporter and has been shown to be important for yeast co-cultivation with lactic acid bacteria (Velasco et al. 2004; Kapetanakis et al. 2021).

While the transcriptional response to DLA has not been explored before, there are studies on transcriptional changes in response to LLA (Kawahata et al. 2006; Abbott et al. 2008). We have compared the changes in the genes differentially expressed in response to 45 mM LLA according to our data and each of the two studies and found substantial positive correlation (Supplemental Fig. S4), even though all experimental designs were different.

In the case of the work by Kawahata et al. (2006), experimental exposures were performed in shake flask cultures with 0.3% LLA (about 33 mM), and the S288C strain (parental to BY472) was used. Two experimental designs were used. First, acid shock was performed by pre-growing the cultures to the optical density at 660 nm (OD660) of 1.0 and exposing them to LLA for 30 min. The second design, acid adaptation, involved diluting overnight cultures to the OD660 = 0.1 in the media with LLA and growing until OD660 reached 1. The number of overlapping DEGs was quite low in both cases (five genes), but the changes in the transcription of these genes were mostly similar to our results (Supplemental Fig. S4a, b).

In the study by Abbott et al. (2008), chemostat cultures of the CEN.PK 113-7D strain (not closely related to S288C and BY4742) were subjected to quite high concentrations of LLA, namely, 500 mM LLA at pH 3 and 900 mM LLA at pH 5. The lists of DEGs shared in our results and these data were larger (over 30 genes in each case), and the correlation of our 45 mM-LLA exposure was much higher with 500 mM LLA than with 900 mM LLA.

We were particularly interested in the D-lactate metabolism genes *DLD1* and *DLD3*. Originally, both of these genes were shown to code for a mitochondrial and cytoplasmic D-lactate dehydrogenases, respectively (Lodi and Ferrero 1993; Chelstowska et al. 1999). According to our results (Fig. 2a; Supplemental Table S3), *DLD1* was upregulated in response to 5 mM DLA (fold change = 1.92 and adjusted *p* = 0.04), as well as in response to 45 mM DLA (fold change = 2.76 and adjusted *p* = 0.0001) and had a similar trend in response to 45 mM LLA, even though the difference did not reach statistical significance (fold change = 1.85 and adjusted *p* = 0.06). Interestingly, Abbott et al. (2008) also found upregulation of *DLD1* to the highest LLA concentration used in their experimental design, 900 mM (fold change = 4 and adjusted *p* = 0.0002). This non-stereo-specific regulation could be interesting to explore further.

While the Dld1 enzyme is the major D-lactate dehydrogenase, Dld3 is a minor D-lactate dehydrogenase and mostly acts as a transhydrogenase coupling D-2-hydroxyglutarate degradation to DLA synthesis (Lodi and Ferrero 1993; Chestowska et al. 1999; Becker-Kettern et al. 2016). In our experiment, *DLD3* was only upregulated in response to 45 mM DLA but not 5 mM DLA or lower concentrations and did not react to DLS, also corroborating the idea of its very minor role as a D-lactate dehygrogenase. It is possible that this protein acts at high DLA concentrations to prevent cell damage by low pH.

In general, our data enrich our understanding of the yeast transcriptome-wide response to LLA and provide the first description of the response to DLA. We found that even though the response to different stereoisomers of lactic acid had quite significant similarities to the response to other weak acids tested previously and largely dependent on pH, there are large differences between DLA and LLA responses, which probably reflect the difference in their role in yeast biology. The role of pH in the DLA response highlights the importance of controlling and optimizing lactic acid production in yeast under neutralizing and non-neutralizing conditions separately, which is also corroborated by a recent work on a recombinant yeast strain with improved lactic acid tolerance and lactic acid yield under non-neutralizing conditions (Yamada et al. 2021).

## Supplementary Information

Below is the link to the electronic supplementary material.

Supplementary file1 (PDF 649 KB)

Supplementary file2 (XLSX 14 KB)

Supplementary file3 (XLSX 4.05 MB)

Supplementary file4 (XLSX 25.2 KB)

## Data Availability

The datasets generated during and analyzed during the current study are available in the GitHub repository, https://github.com/drozdovapb/S_cerevisiae_lactate_transcriptome and the NCBI GEO repository under the accession number GSE231937.
