# Supplementary material for: Transcriptional response of Saccharomyces cerevisiae to lactic acid enantiomers
Source: Appl Microbiol Biotechnol. 2024 Jan 13;108:121. doi: 10.1007/s00253-023-12863-z (PMC10787881; doi:10.1007/s00253-023-12863-z)
Supplement: Supplementary file 1 [file 253_2023_12863_MOESM1_ESM.pdf]

Applied Microbiology and Biotechnology

Supplemental Material for:

Transcriptional response of *Saccharomyces cerevisiae* to lactic acid enantiomers

Polina Drozdova[1,2], Anton Gurkov[1,2], Alexandra Saranchina[1], Anastasia Vlasevskaya[1], Elena Zolotovskaya[1], Elizaveta Indosova[1], Maxim Timofeyev[1], Ekaterina Borvinskaya[1]

[1] Irkutsk State University, Karl-Marx str. 1, Irkutsk 664025, Russian Federation

[2] Baikal Research Centre, Rabochaya str. 5V, Irkutsk 664011, Russian Federation

Address correspondence to:

Polina Drozdova drozdovapb@gmail.com; Ekaterina Borvinskaya borvinska@gmail.com

Phone +7-3952-24-30-77; fax +7-3952-34-00-07.

Supplemental figures

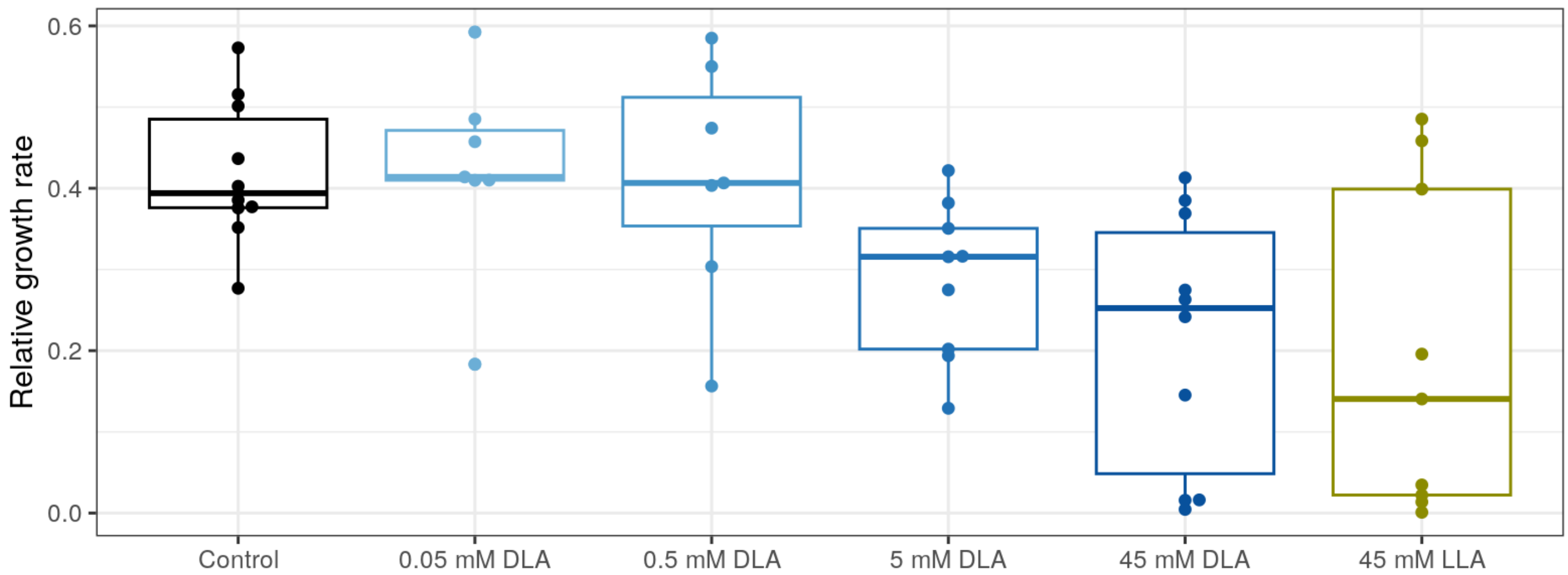

Fig. S1 The relative growth rate (the difference between logarithmic (base 2) final OD600 and initial OD600 divided over incubation time in hours) of cultures cultivated for 3-4 hours in the media with the indicated concentrations of DLA or LLA

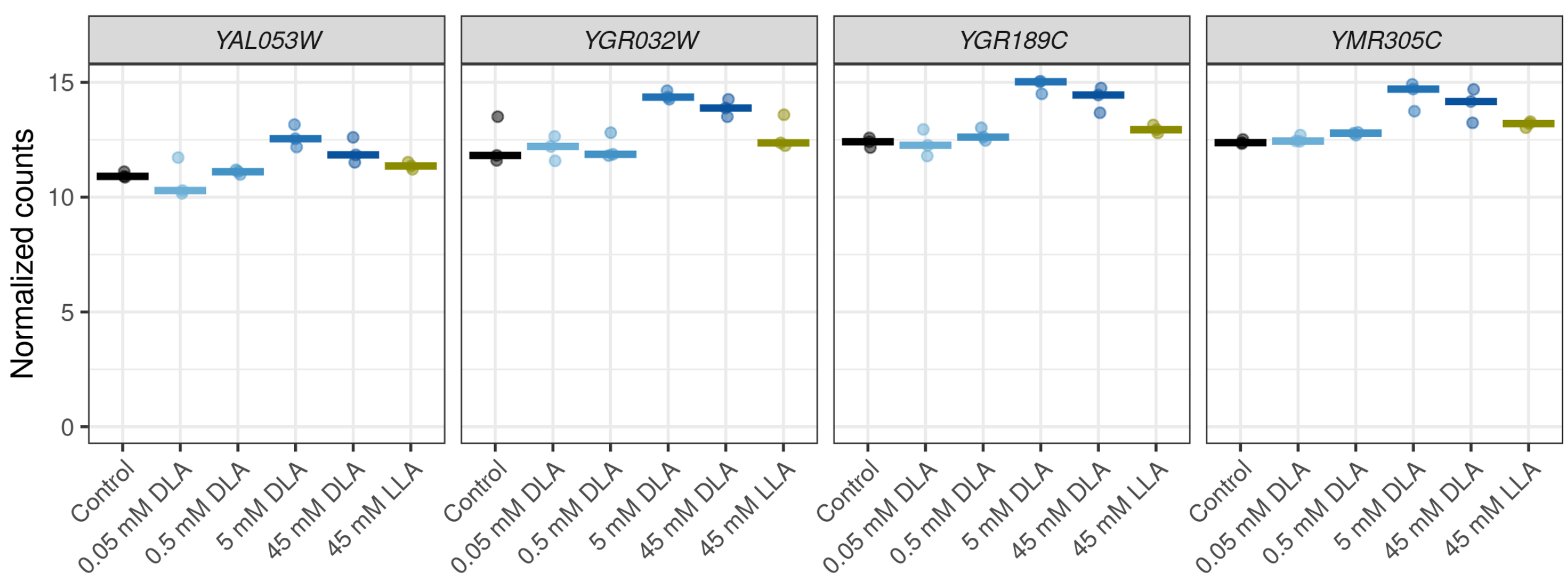

Fig. S2 Examples of DE genes associated with the cell wall biosynthesis. Shown are the four top DEGs for 5 mM DLA. The vertical axis shows logarithmic (base 2) normalized expression counts of the genes

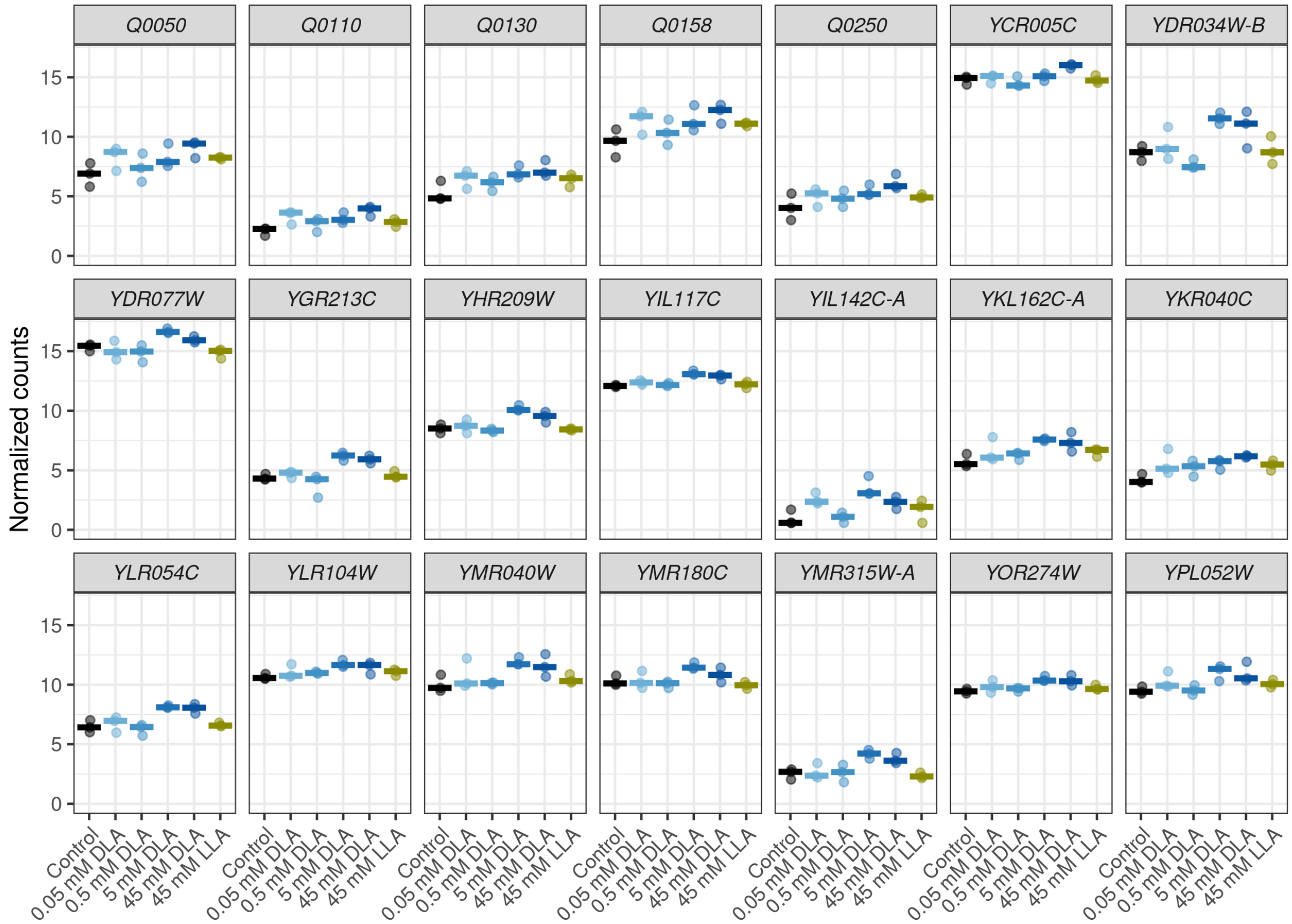

Fig. S3 Expression levels of the genes from cluster 1 (see Fig. 3). The vertical axis shows logarithmic (base 2) normalized expression counts of the genes. The vertical axis shows logarithmic (base 2) normalized expression counts of the genes

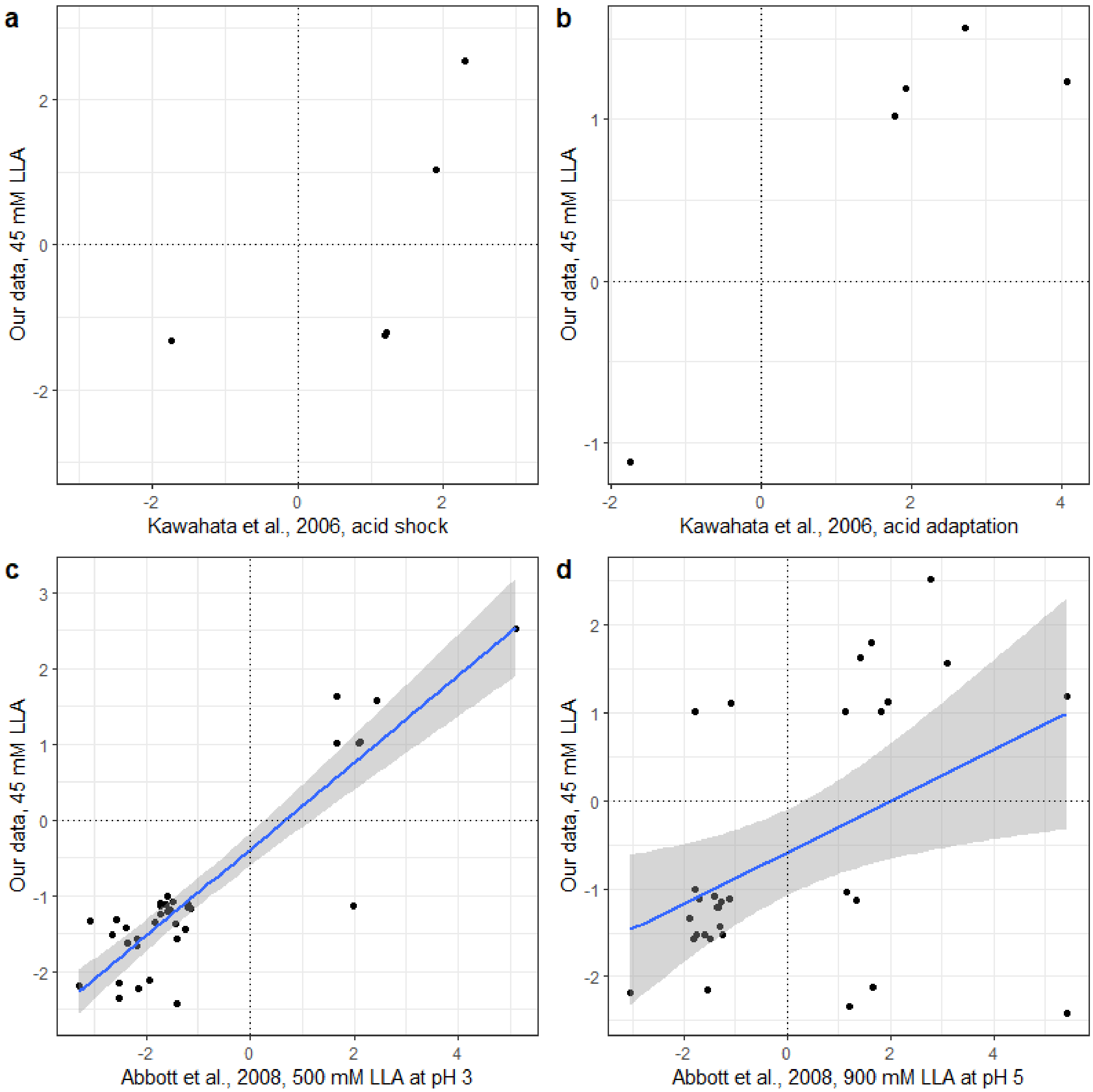

Fig. S4 Correlation between DEG expression profiles in response to 45 mM LLA obtained in this work ('our data') and the data from different experimental designs: Kawahata et al., 2006 (a, b) and Abbott et al., 2008 (c, d). Pearson's correlation coefficient equaled 0.66, 0.92, 0.90, and 0.44, respectively

Supplementary tables

Table S1 Primers used for qPCR

Target gene	Forward primer	Reverse primer	Product length, bp	Amplification efficiency, %	Source
ACT1	AGAGTTGCCCCAGAAGAACA	GGCTTGGATGGAAACGTAGA	132	88	Cankorur-Cetinkaya et al. (2012)
AQR1	GACTGCAAGGTGGGATTGGA	TCGGCGCTCTGTTGATGAAT	155	96	this work
CDC19	CAGAGGTGACTTGGGTATTG	GGTTGGTCTTGGGTTGTAAG	145	91	Cankorur-Cetinkaya et al. (2012)
DLD3	AGAGATTAAACGCAGCCGGT	GCTCAGCACTGATGGAACCT	195	91	this work
FIT2	ACCAAGACCGTCACTCAAGC	CAATGCACCGACACTTGCTC	129	99	this work
YPS3	CAAGTACGAGGGCCAACTGT	TGAATCGAGTAGGGCTGGGA	166	100	this work

Table S2 (in a separate file) Yeast growth characteristics. sample_id, sample ID consisting of the date experiment date in the yyyymmdd format; condition, experimental condition; OD600_init, initial optical density at 600 nm after diluting the overnight culture in each medium; OD600_final, final optical density at 600 nm after 3-4 hours of inclubation; Time, h, incubation time; and RGR2, relative growth rate calculated as the difference between logarithmic (base 2) final OD600 and initial OD600 divided over incubation time in hours. Sheet 'Data_growth_inhibition' contains the data used for Supplemental Fig. S1, while sheet 'Data_pH_compensation' contains the data used for Fig. 4a

Table S3 (in a separate file) Differentially expressed genes (log_2 fold change > 1 and adjusted *p*-value < 0.05) identified with DESeq2 in each comparisons (sheet names). baseMean, mean expression; lfcSE, standard error of the log_2 fold change; stat, Wald statistics; padj, *p*-value adjusted according to the Benjamin-Hochberg procedure. Raw data are available from the GEO database (GSE231937)

Table S4 Manuscripts enriched in the DEGs identified with YeastMine

Comparison	Overrepresented manuscripts in YeastMine (title and doi)
5 mM DLA, upregulated DEGs	• The high osmotic response and cell wall integrity pathways cooperate to regulate transcriptional responses to zymolyase-induced cell wall stress in *Saccharomyces cerevisiae*. https://doi.org/10.1074/jbc.M808693200<br>• Characterization of the transcriptional response to cell wall stress in *Saccharomyces cerevisiae*. https://doi.org/10.1002/yea.1109<br>• The cell wall and the response and tolerance to stresses of biotechnological relevance in yeasts. https://doi.org/10.3389%2Ffmicb.2022.953479<br>• The CWI Pathway: Regulation of the Transcriptional Adaptive Response to Cell Wall Stress in Yeast. https://doi.org/10.3390%2Fjof4010001<br>• Genome-wide analysis of gene expression regulated by the yeast cell wall integrity signalling pathway. https://doi.org/10.1046/j.1365-2958.1999.01667.x<br>• Conserved processes and lineage-specific proteins in fungal cell wall evolution. https://doi.org/10.1128/ec.00044-07

	• Adaptation of Saccharomyces cerevisiae to high hydrostatic pressure causing growth inhibition. https://doi.org/10.1016/j.febslet.2005.03.100<br>• Up-regulation of genes encoding glycosylphosphatidylinositol (GPI)-attached proteins in response to cell wall damage caused by disruption of FKS1 in *Saccharomyces cerevisiae*. https://doi.org/10.1007/s004380000285<br>• Genomic binding sites of the yeast cell-cycle transcription factors SBF and MBF. https://doi.org/10.1038/35054095<br>• Production of polyunsaturated fatty acids in yeast *Saccharomyces cerevisiae* and its relation to alkaline pH tolerance. https://doi.org/10.1002/yea.1659<br>• Cell wall construction in *Saccharomyces cerevisiae*. https://doi.org/10.1002/yea.1349<br>• Gene duplication and co-evolution of G1/S transcription factor specificity in fungi are essential for optimizing cell fitness. https://doi.org/10.1371/journal.pgen.1006778<br>• Differential gene expression signatures for cell wall integrity found in chitin synthase II (chs2Delta) and myosin II (myo1Delta) deficient cytokinesis mutants of *Saccharomyces cerevisiae*. https://doi.org/10.1186/1756-0500-2-87
5 mM DLA, downregulated DEGs	• A comprehensive analysis of gene expression profiles in a yeast N-glycosylation mutant. https://doi.org/10.1006/bbrc.2001.5457<br>• Mitochondrial Compartmentalization Confers Specificity to the 2-Ketoacid Recursive Pathway: Increasing Isopentanol Production in *Saccharomyces cerevisiae*. https://doi.org/10.1021/acssynbio.9b00420<br>• Yeast mating: a model system for studying cell and nuclear fusion. https://doi.org/10.1007/978-1-59745-250-2_1<br>• Quantitative and dynamic analyses of G protein-coupled receptor signaling in yeast using Fus1, enhanced green fluorescence protein (EGFP), and His3 fusion protein. https://doi.org/10.1021/bp0601387<br>• A focus on yeast mating: From pheromone signaling to cell-cell fusion. https://doi.org/10.1016/j.semcdb.2022.02.003<br>• Regulation of mating and filamentation genes by two distinct Ste12 complexes in *Saccharomyces cerevisiae*. https://doi.org/10.1128/mcb.02053-05
45 mM DLA, upregulated DEGs	• Copper supplementation increases yeast life span under conditions requiring respiratory metabolism. https://doi.org/10.1016/j.mad.2006.10.003
5 mM DLA, downregulated DEGs	• A comprehensive analysis of gene expression profiles in a yeast N-glycosylation mutant. https://doi.org/10.1006/bbrc.2001.5457<br>• Repressors Nrg1 and Nrg2 regulate a set of stress-responsive genes in *Saccharomyces cerevisiae*. https://doi.org/10.1128/ec.4.11.1882-1891.2005
45 mM LLA, upregulated DEGs	• *Saccharomyces cerevisiae* adaptation to weak acids involves the transcription factor Haa1p and Haa1p-regulated genes. https://doi.org/10.1016/j.bbrc.2005.09.010<br>• Genomic expression program involving the Haa1p-regulon in *Saccharomyces cerevisiae* response to acetic acid. https://doi.org/10.1089/omi.2010.0048<br>• Nuclear localization of Haa1, which is linked to its phosphorylation status, mediates lactic acid tolerance in *Saccharomyces cerevisiae*. https://doi.org/10.1128/aem.04241-13<br>• MFS transporters required for multidrug/multixenobiotic (MD/MX) resistance in the model yeast: understanding their physiological function through post-genomic approaches. https://doi.org/10.3389/fphys.2014.00180<br>• Siderophore-iron uptake in *Saccharomyces cerevisiae*. Identification of ferrichrome and fusarinine transporters. https://doi.org/10.1074/jbc.m001456200<br>• Identification of a DNA-binding site for the transcription factor Haa1, required for *Saccharomyces cerevisiae* response to acetic acid stress. https://doi.org/10.1093/nar/gkr228
45 mM LLA, downregulated DEGs	• A complete mass-spectrometric map of the yeast proteome applied to quantitative trait analysis. https://doi.org/10.1038/nature11835<br>• Genome-wide responses to mitochondrial dysfunction. https://doi.org/10.1091/mbc.12.2.297<br>• Gene regulatory changes in yeast during life extension by nutrient limitation. https://doi.org/10.1016/j.exger.2010.02.008<br>• Role of transcriptional regulation in controlling fluxes in central carbon metabolism of *Saccharomyces cerevisiae*. A chemostat culture study. https://doi.org/10.1074/jbc.m309578200<br>• Photocrosslinking and click chemistry enable the specific detection of proteins interacting with phospholipids at the membrane interface. https://doi.org/10.1016/j.chembiol.2008.11.009

	• Protein phosphorylation in mitochondria --a study on fermentative and respiratory growth of *Saccharomyces cerevisiae*. https://doi.org/10.1002/elps.200900759<br>• Effect of calorie restriction on the metabolic history of chronologically aging yeast. https://doi.org/10.1016/j.exger.2009.06.001<br>• Manganese is the link between frataxin and iron-sulfur deficiency in the yeast model of Friedreich ataxia. https://doi.org/10.1074/jbc.m511649200<br>• Proteome analysis of aerobically and anaerobically grown *Saccharomyces cerevisiae* cells. https://doi.org/10.1016/j.jprot.2008.11.012<br>• Rsf1p is required for an efficient metabolic shift from fermentative to glycerol-based respiratory growth in *S. cerevisiae*. https://doi.org/10.1002/yea.1655<br>• Central carbon metabolism of *Saccharomyces cerevisiae* in anaerobic, oxygen-limited and fully aerobic steady-state conditions and following a shift to anaerobic conditions. https://doi.org/10.1111/j.1567-1364.2007.00234.x<br>• Analysis of the vacuolar luminal proteome of *Saccharomyces cerevisiae*. https://doi.org/10.1111/j.1742-4658.2007.05959.x
45 mM DLA *vs*. 45 mM LLA, upregulated DEGs	• Characterization of the transcriptional response to cell wall stress in *Saccharomyces cerevisiae*. https://doi.org/10.1002/yea.1109

Table S5. qPCR results. Sample name, internal sample ID (RT* = no-RT control; K* = control; DLS45* = 45 mM DLS; empty = RNAse-free water); Target name, the name of the gene, fragment of which is being amplified; Task, sample type (NTC = no template control; Unknown = with DNA or RNA); Reporter = reporter dye (SYBR Green); CT = cycle threshold; CT Mean = mean CT between technical replicates; CT SD = standard deviation of the CT values between technical replicates; Tm = melting temperature peak(s)

References mentioned in the Supplemental Materials

Abbott DA, Suir E, van Maris AJA, Pronk JT (2008) Physiological and transcriptional responses to high concentrations of lactic acid in anaerobic chemostat cultures of *Saccharomyces cerevisiae*. Appl Environ Microbiol 74:5759–5768. doi: 10.1128/AEM.01030-08

Cankorur-Cetinkaya A, Dereli E, Eraslan S, Karabekmez E, Dikicioglu D, Kirdar B (2012) A novel strategy for selection and validation of reference genes in dynamic multidimensional experimental design in yeast. PloS One 7: e38351. doi: 10.1371/journal.pone.0038351

Kawahata M, Masaki K, Fujii T, Iefuji H (2006) Yeast genes involved in response to lactic acid and acetic acid: acidic conditions caused by the organic acids in *Saccharomyces cerevisiae* cultures induce expression of intracellular metal metabolism genes regulated by Aft1p. FEMS Yeast Res 6:924–936. doi: 10.1111/j.1567-1364.2006.00089.x
